# Multi-responsive nanofibers composite gel for local drug delivery to inhibit recurrence of glioma after operation

**DOI:** 10.1186/s12951-021-00943-z

**Published:** 2021-07-03

**Authors:** Yufu Zhu, Jun Jia, Gang Zhao, Xuyang Huang, Lansheng Wang, Yongkang Zhang, Long Zhang, Naveena Konduru, Jun Xie, Rutong Yu, Hongmei Liu

**Affiliations:** 1 Institute of Nervous System Diseases, Xuzhou Medical University, Xuzhou, 221002 China; 2 Department of Neurosurgery, Affiliated Hospital of Xuzhou Medical University, Xuzhou, 221002 China; 3 Department of Neurosurgery, The Third People’s Hospital Affiliated of Xuzhou Medical University, Xuzhou, 221002 China; 4 Institute of International Education, Xuzhou Medical University, Xuzhou, 221002 China; 5 School of Life Science, Jiangsu Normal University, Xuzhou, 221116 China

**Keywords:** Local drug delivery, Glioma, Hydrogel, Operation, Recurrence

## Abstract

**Background:**

The postoperative recurrence of malignant gliomas has presented a clinical conundrum currently. Worse, there is no standard treatment for these recurrent tumours. Therefore, novel promising methods of clinical treatment are urgently needed.

**Methods:**

In this study, we synthesized reactive oxygen species (ROS)-triggered poly(propylene sulfide)60 (PPS60) mixed with matrix metalloproteinases (MMPs)-responsive triglycerol monostearate (T) lipids and TMZ. The mixed solution could self-assemble at 50 ℃ to generate hydrogels with MMPs- and ROS-responsiveness. We explored whether the T/PPS + TMZ hydrogel could achieve the MMP- and ROS-responsive delivery of TMZ and exert anti-glioma regrowth effects in vitro and in vivo. These results demonstrated that the T/PPS + TMZ hydrogel significantly improved the curative effect of TMZ to inhibit postsurgical recurrent glioma.

**Results:**

The results confirmed the responsive release of TMZ encapsulated in the T/PPS + TMZ hydrogel, and the hydrogel showed excellent performance against glioma in an incomplete glioma operation model, which indicated that the T/PPS + TMZ hydrogel effectively inhibited the growth of recurrent glioma.

**Conclusion:**

In summary, we successfully developed injectable MMPs- and ROS-responsive hydrogels that could achieve the sustained release of TMZ in the surgical cavity to inhibit local recurrent glioma after surgery.

**Graphic abstract:**

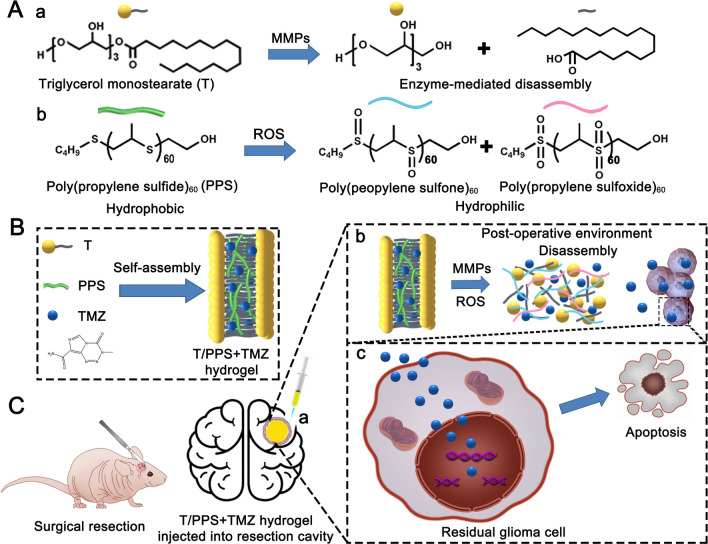

**Supplementary Information:**

The online version contains supplementary material available at 10.1186/s12951-021-00943-z.

## Background

Glioma is the most common type of primary tumour in the brain and is derived from the nerve epithelium [1, 2]. Although therapies against malignant gliomas, including surgery, radiotherapy and chemotherapy, have been widely used, the therapeutic effect remains poor [3–6]. The median survival of malignant glioma patients is less than 14.6 months [7, 8]. The hardest problem in treating glioma is postoperative recurrence. Complete resection is deemed impossible in high-grade gliomas, and residual glioma cells contribute to postoperative glioma recurrence [9]. Clinical studies found that 80–90% of recurrent gliomas occur within 2 cm of the original region [8, 10, 11]. Decreasing glioma recurrence caused by residual tumour cells has become an important topic in clinical research and practice.

Currently, there are no specific cures for recurrent gliomas. TMZ is still a first-line chemotherapeutic for the clinical treatment of recurrent gliomas [12–14]. However, the therapeutic efficacy of TMZ is often limited by several factors, including its short half-life in vivo, rapid decomposition, blood–brain barrier (BBB) permeability, and chemoresistance induced by *O*6-methylguanine-DNA methyltransferase (MGMT) [13, 15–17].

To overcome the above problems, scholars have attempted to deliver TMZ directly to tumour regions in situ. Local TMZ delivery can avoid the systemic circulation of drugs, reduce the toxicity to normal tissues, provide localized sustained release of drugs, and thereby increase the amounts of drugs in the tumour site [18–20]. Injectable drug-loaded hydrogels, as a local drug delivery method, have attracted much attention in glioma therapies because they could bypass the BBB and act directly on the tumour regions to increase local drug concentrations while minimizing the adverse effects of systemic exposure to the drug [19, 21–25].

Hydrogels are polymer network systems with water as the dispersion medium that can be fabricated by UV irradiation, introducing irreversible covalent bonds, or by self-assembly through chemical reactions [26, 27]. The mechanical properties of hydrogels can be continuously regulated by controlling the ratio of water. In addition, hydrogels possess the advantages of good biocompatibility, non-cytotoxicity, and low price, and they show broad prospects for application in the field of cancer treatment [28, 29]. Drug delivery systems based on hydrogels have been widely used in preclinical studies [25, 30–33]. However, due to the lack of balance among various considerations, this very promising strategy did not work as expected. To date, only Gliadel® has been approved by the FDA for clinical treatment, and has exhibited limited efficacy and many adverse effects compared to standard chemotherapy [34].

**Fig. 1:**
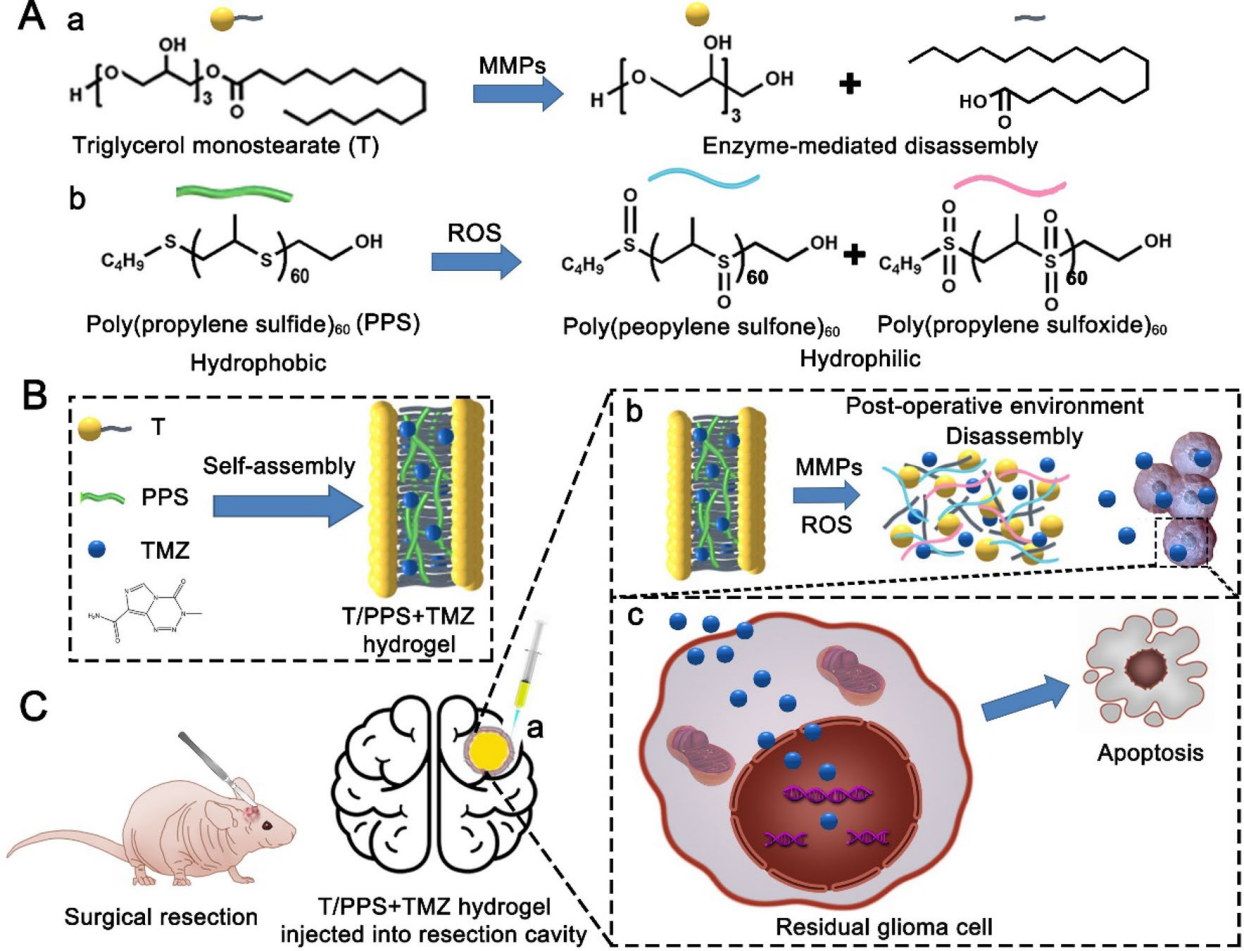
Structure and application of the MMPs- and ROS-responsive T/PPS + TMZ hydrogel for inhibiting recurrent glioma after surgery. **A** The structure of T and MMPs-responsive ability (**a**). The structure of PPS60 and ROS-responsive ability (**b**). **B** Schematic representation of the self-assembly of T, PPS60 and TMZ to form the T/PPS + TMZ hydrogel. **C** T/PPS + TMZ hydrogel injected into the surgical cavity (**a**). TMZ was released from the T/PPS + TMZ hydrogel in a postoperative environment (**b**). Released TMZ entered residual glioma cells to induce the apoptosis of glioma cells (**c**)

In this study, we developed a biodegradable, dual-responsive (ROS- and MMPs-) hydrogel to achieve local TMZ delivery (T/PPS + TMZ). T lipid-covered PPS60 hydrogels were prepared to load TMZ and implanted into the surgical cavity (Fig. 1). The T/PPS + TMZ hydrogel possessed the following features: (1) T lipids were present in the outside layer of the T/PPS + TMZ hydrogel with MMP responsiveness, while ROS-responsive PPS60 was present in the inside layer; (2) the T/PPS + TMZ hydrogel showed a semisolid nature with a degree of fluidity; and (3) released TMZ entered into gliomas to kill residual glioma cells. In vivo, the T/PPS + TMZ hydrogel effectively reduced the proliferation of residual glioma cells and enhanced the efficiency of TMZ. In summary, our research presented a promising strategy to inhibit tumour recurrence, which enabled further exploration.

## Methods

### Materials

d-Luciferin potassium salt, TMZ, recombinant human MMP2 and MMP9 were obtained from Dalian Meilun Biotech Co., Ltd. (Dalian, China). The Live-Dead Assay Kit was purchased from Jiangsu Keygen Biotech Co., Ltd. (Nanjing, China). Eater-terminated poly d,l-lactic-co-glycolic acid (PLGA), propylene sulfide, and 2-iodoethanol were purchased from Sigma-Aldrich.

### Mice and cells

Male mice (C57BL/6 and BALB/c nude, 6–8 weeks) were purchased from HFK Bioscience Co., Ltd. (Beijing, China). The animal research was approved by the Animal Care and Use Committee of Xuzhou Medical University. The glioma cell lines (GL261, C6 and U87) came from the Shanghai Cell Bank, Chinese Academy of Sciences (Shanghai, China). C6-GFP-Luci cells were transfected with the luciferase gene. All cells were cultured in DMEM (Gibco, Carlsbad, CA, USA) supplemented with 10% foetal bovine serum with 1% penicillin sodium and streptomycin sulfate.

### Synthesis of PPS60 and characteristics of hydrogels

PPS_60_-OH was synthesized according to the previous literature with a few modifications [35]. In a dried and nitrogen-flushed RB flask, 1,8-diazabicyclo[5.4.0]undec-7-ene (DBU) (258 mg, 1.5 mmol) in dry THF was degassed for 30 min, and the reaction mixture temperature was lowered to 0 °C. To this flask, a degassed solution of 1-butane thiol (45 mg, 0.5 mmol) was added dropwise and allowed to react for 30 min. Later, degassed propylene sulfide (2.96 g, 40 mmol) monomer was added to the reaction mixture, and the temperature was maintained at 0 °C for 2 h. The reaction was quenched by the addition of 2-iodoethanol (172 mg, 1 mmol) and stirred overnight at room temperature. The next day, the polymer solution was filtered to remove precipitated salt and further purified by three precipitations into cold methanol before vacuum-drying to yield a colourless viscous polymer. ^1^H NMR (400 MHz, CDCl_3_): δ 3.75 (s, 3H), 3.48 (q, *J* = 7.0 Hz, 2H), 3.20–2.74 (m, 116H), 2.70–2.58 (m, 54H), 2.54 (dd, *J* = 8.3, 4.0 Hz, 3H), 1.58 (t, *J* = 7.5 Hz, 5H), 1.49–1.10 (m, 176H), 0.92 (t, *J* = 7.3 Hz, 3H). GPC was performed using a water system equipped with a refractive index and a photodiode array detector, with DMF as the eluent (0.5 mL min^−1^, 40 °C) and polystyrene standards used for calibration. Mn_(GPC)_ = 4.10 × 10^3^, Mw_(GPC)_ = 4.95 × 10^3^, Mw/Mn = 1.21.

Then, 100 mg T, 16 mg PPS and 4 mg TMZ were placed in a glass vial, and 200 µL DMSO and 800 µL water were added. The mixed solution was added to an MW = 3500 Da dialysis bag and dialyzed for 24 h to remove DMSO. Then, this glass vial was placed in a 50 °C water bath and heated until the mixture was completely dissolved. The vial was then removed and cooled to room temperature. When the inverted vial was observed to have no gravitational flow, the gelation of the T/PPS hydrogel was completed. T/PLGA/TMZ, T/PLGA/DiR and T/PPS/DiR were prepared by the same method. The concentration of DiR was 100 µg mL^−1^.

The hydrogel was imaged using a high-resolution transmission electron microscope (FEI Tecnai G2 Spirit Bio TWIN, FEI, Hillsboro, OR, USA). The hydrogel was prepared as described in the previous section and diluted by a factor of 20 with dd water. The sample was dropped on a copper grid coated with carbon film, stained for 1 min, and air-dried at room temperature. Finally, it was observed by TEM and photographed.

The rheological properties of the hydrogel at 37 °C were evaluated with a dynamic rheometer (TA Instruments, USA). Data on the storage modulus (G′) and loss modulus (G″) were recorded with a dynamic strain frequency sweep (0.1–100 Hz, strain 5%) measurement frequency scanning range from 0.02 to 100 rad/s.

### Disassembly of DiR-loaded T/PPS and T/PLGA hydrogel in vitro

Next, 50 µL T/PPS/DiR and T/PLGA/DiR hydrogels were added to a 24-well transwell plate and cultured in an oscillating incubator at 37 °C. PBS in wells was changed with fresh PBS with or without MMP2 (100 ng mL^−1^) and H_2_O_2_ (1 mM) on day 0. The hydrogels were incubated in an oscillating incubator at 37 °C again, and the fluorescence intensity was measured on days 0, 1, 2, 3, 5 and 7 by an imaging device (Caliper Life Sciences).

### In vitro MMPs- and ROS-responsive drug release

100 µL T/PPS + TMZ hydrogel and T/PLGA + TMZ hydrogel were placed in a centrifuge tube, containing PBS buffer without or with one of the following reagents: MMP2 (100 ng mL^−1^), MMP9 (100 ng mL^−1^) and H_2_O_2_ (1 mM). The drug concentration was determined as we previously reported [36]. In some tubes, MMP2 or MMP9 inhibitors were added. Fresh MMP2, MMP9 or MMP2, MMP9 + MMP2, and MMP9 inhibitors were added on days 1, 3 and 5. The tubes were incubated at 37 °C with shaking (70 rpm). A 100 μL PBS reservoir was taken at different time points, the concentration of TMZ was analysed by HPLC (Shimadzu, LC-10AT/sd-10a, column: Hyersil ODS C18 4.6 200 mm, particle size 5 m), and a release profile was drawn according to the protocol.

The standard curve of TMZ was y = 0.1158x − 0.1756, R^2^ = 0.9963.

### In vitro biocompatibility

C6 or U87 cells (6 × 10^3^) were placed in 96-well plates and cultured for 24 h. Then, the T/PLGA and T/PPS hydrogel soaking solutions were replaced with medium. After incubation for 24 h, the cytotoxicity of the hydrogel was tested by MTT assay. The absorbance at 570 nm was measured to obtain the results. Alternatively, C6 cells were cultured on 24-well plates containing 5 × 10^4^ cells. After 24 h, PBS or hydrogel immersion solutions were added to the medium. Cells were stained with a live-dead assay kit after 72 h and observed by fluorescence microscopy (Olympus, Takachiho, Japan).

### Disassembly of DiR-loaded T/PPS and T/PLGA hydrogel in vivo

A GL261 glioma surgical model was constructed, and the tumour-bearing mice were divided into two groups: Group 1: 10 µL T/PLGA + DiR hydrogel was injected into the resection cavity (n = 3); Group 2: 10 µL T/PPS + DiR hydrogel was injected into the resection cavity (n = 3). The fluorescence intensity was measured at days 0, 1, 2, 3, 5 and 7.

### Determination of MMPs and ROS in the postoperative environment

The expression of MMPs (MMP2 and MMP9) in the postoperative environment was determined using ELISA kits purchased from Laitian (Nanjing, China). Incomplete operations on GL261 glioma-bearing mice were performed, and the residual tumour tissue was collected 1 h later. As a control, tumour tissues from mice that did not undergo surgery were collected. Then, ELISA kits were used according to the manufacturer’s instructions.

The expression of ROS was detected by dihydroethidium (DHE) dye. Briefly, samples were collected by the same methods and sectioned. The sections were incubated with DHE for 30 min at 37 °C and then mounted using DAPI and coverslips. The sections were observed using a fluorescence microscope. Fluorescence intensities were quantified with ImageJ software.

### GL261, C6-GFP-Luci and U87 glioblastoma orthotopic models and antitumour efficacy after peritumoural administration of the hydrogels in the resection cavity

Glioma resection was performed as described in our previous research with a few adjustments [37, 38]. Tumour-bearing mice were removed and divided into four groups (n = 9) on day 12 after tumour inoculation: Group 1: PBS (no treatment); Group 2: blank T/PPS hydrogel (10 µL) was injected into the surgical cavity; Group 3: T/PLGA + TMZ hydrogel (10 µL) was injected into the surgical cavity; Group 4: T/PPS + TMZ hydrogel (10 µL) was injected into the surgical cavity. On the 40th day after glioma transplantation, mice in each group (n = 3) were euthanized and sacrificed, and sections of the tumours were stained with H&E and analysed by immunofluorescence histochemical analysis (TUNEL expression).

Animals with C6-GFP-Luci tumours were divided into four groups (n = 9) on day 8 after tumour inoculation: Group 1: PBS (no treatment); Group 2: blank T/PPS hydrogel (10 µL) was injected into the surgical cavity; Group 3: T/PLGA + TMZ hydrogel (10 µL) was injected into the surgical cavity; Group 4: T/PPS + TMZ hydrogel (10 µL) was injected into the surgical cavity. Tumour growth was tested and quantified by bioluminescence intensity with the IVIS kinetic imaging system on the 7th, 14th and 21st days after glioma implantation.

### Toxicity evaluation

Tumour-bearing mice in different groups (n = 3) were sacrificed. The blood and the main organs, including the heart, liver, spleen, lung and kidney, were collected. The main organs were stained with H&E. Plasma was separated by centrifugation and analysed for hepatorenal toxicity by a cobas 8000 modular analyzer.

### Statistical analysis

All experiments were performed at least 3 times, and the results are presented as the mean ± SD. Statistical analysis of the data was performed with Student’s t-tests or one-way ANOVAs, and P values < 0.05 were considered to be statistically significant (*P < 0.05, **P < 0.01, ***P < 0.001).

## Results

### Synthesis and characterization of T/PPS + TMZ hydrogel

**Fig. 2:**
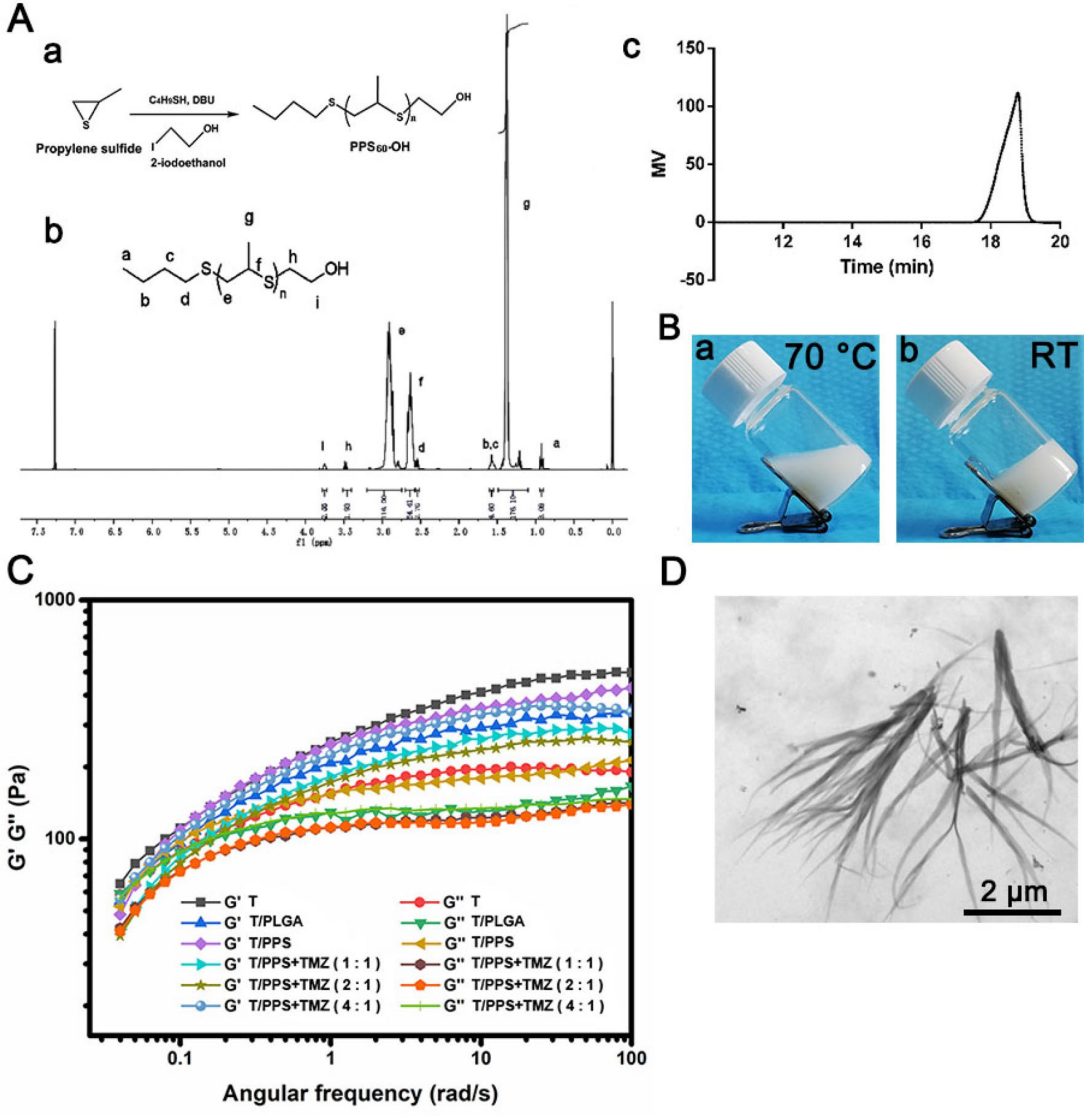
Preparation and characterization of T/PPS + TMZ hydrogels. **A** Synthesis route (**a**) and ^1^H NMR of PPS60-OH (**b**). GPC traces of PPS60-OH with DMF as the elute (**c**). **B** Gelation of the T/PPS + TMZ hydrogel before (**a**) and after cooling (**b**). **C** Rheological analysis of the hydrogels. **D** TEM images of T/PPS + TMZ hydrogel. Scale bar = 2 µm

ROS-responsive PPS_60_ was synthesized, and the structure of PPS_60_ was confirmed by ^1^H NMR (Fig. 2A(a, b)). The molecular weight of PPS was 4.1 × 10^3^, and the polydispersity (PDI) was 1.21, as determined by GPC (Fig. 2A(c)).

The MMP_S_- and ROS-responsive hydrogel was formed through the self-assembly of T and PPS_60_ in DMSO/water. TMZ was encapsulated into the hydrophobic PPS_60_ layer during the process of T/PPS self-assembly (Fig. 2B). The gelation kinetics of the T/PPS hydrogel were investigated by rheological evaluation (Fig. 2C). Three different PPS and TMZ mass ratios (1: 1, 2: 1, 4: 1, w/w) of the T/PPS + TMZ hydrogel (10% w/v) were prepared and tested by rheological analysis. The values of G′ (storage modulus) and G″ (loss modulus) were markedly increased. The G′ value was approximately three times higher than the G″ value, which was solid evidence of T/PPS hydrogel formation. The PPS and TMZ mixing ratio of 4:1 had the best effect. Thus, the weight ratio of PPS:TMZ 4:1 was used for all subsequent experiments. Next, the surface of the T/PPS + TMZ hydrogel was observed by TEM, which showed higher-order fibrous assemblies (Fig. 2D).

### MMPs- and ROS-triggered disassembly and TMZ release of the T/PPS + TMZ hydrogel

**Fig. 3:**
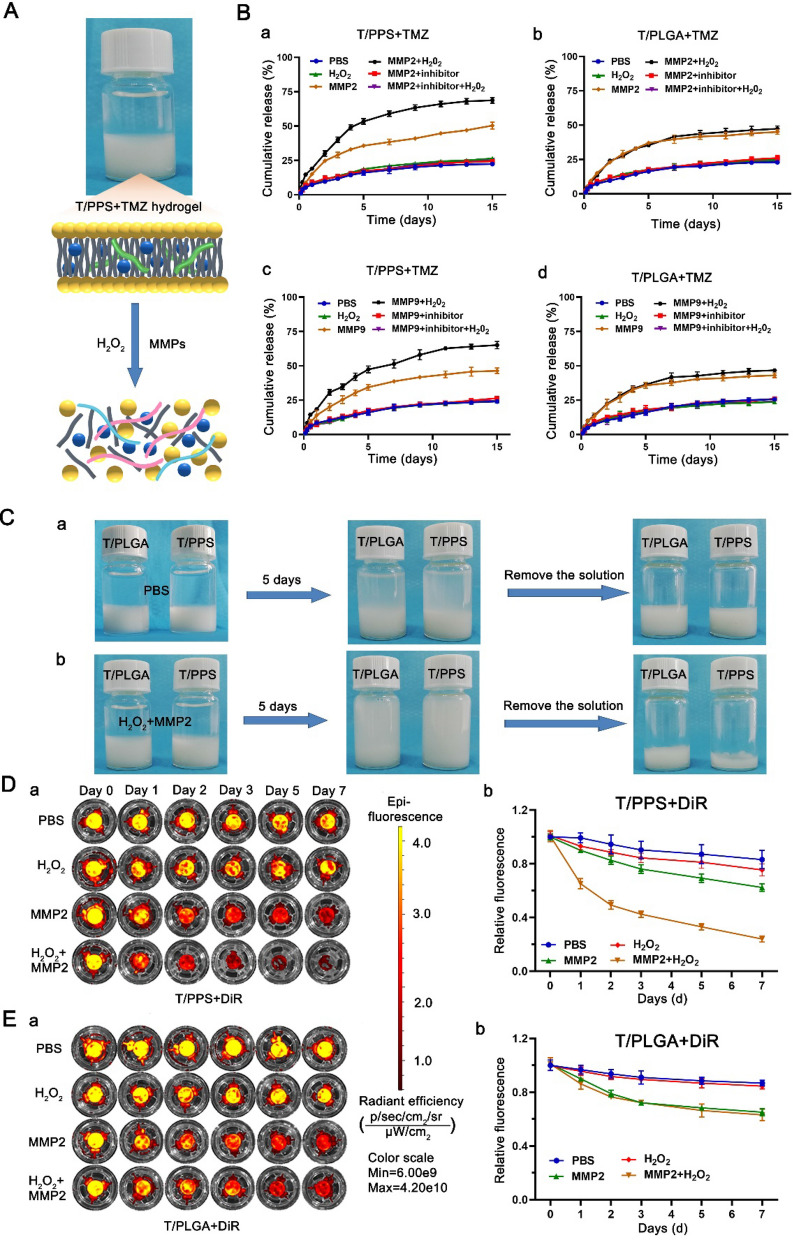
Responsive degradation and TMZ release of the T/PPS + TMZ hydrogel. **A** Schematic illustration of T/PPS + TMZ hydrogel degradation under MMPs and ROS conditions. **B** The TMZ release profile of T/PPS + TMZ hydrogels and T/PLGA + TMZ hydrogels. Fresh MMP2/9 + MMP2/9 inhibitor was added on days 1, 3 and 5. Data are presented as means ± SEM (n = 3). **C** Images of T/PPS hydrogel and T/PLGA hydrogel were immersed in PBS and PBS containing MMP2 and H_2_O_2_ solution for 5 days. The solution was discarded to observe the residual amount of hydrogel. **D** The DiR release profile from T/PPS + DiR hydrogels incubated in PBS without or with MMP2 and H_2_O_2_. **a** DiR fluorescence image. **b** Quantification of fluorescence signals at each time point of the T/PPS + DiR hydrogel. Data are presented as the mean ± SEM (n = 3). **E** The DiR release profile from T/PLGA + DiR hydrogels incubated in PBS without or with MMP2 and H_2_O_2_. **a** DiR fluorescence image. **b** Quantification of fluorescence signals at each time point of the T/PLGA + DiR hydrogel. Data are presented as the mean ± SEM (n = 3)

Postsurgery glioma models were successfully established as previously described [39]. After surgery for glioma, the T/PPS + TMZ hydrogel was injected into a surgical cavity (Additional file 1: Figure S1). MMPs and ROS are overproduced after tissue injury [40–42]. Acute tissue injury was unavoidable during the surgical procedure; therefore, MMPs and ROS were upregulated in the postoperative environment (Additional file 1: Figures S2 and S3). As previously reported, the T hydrogel could degrade in response to MMPs, while PPS showed ROS-responsive activity [35, 43, 44]. Thus, the ability of the T/PPS + TMZ hydrogel to release the encapsulated TMZ in the presence of MMPs and H_2_O_2_ was evaluated. In vitro TMZ release experiments were conducted in the presence of MMPs, H_2_O_2_ or MMP2/MMP9 + H_2_O_2_, mimicking the MMPs and ROS environment in surgical cavity (Fig. 3A). Fresh enzyme was added on days 1, 3 and 5. The addition of MMP2 and MMP9 significantly increased the release of TMZ from the T/PPS + TMZ hydrogel (50.3%, 46.3%) and T/PLGA + TMZ hydrogel (45.0%, 43.0%), indicating that MMPs caused degradation of the T-rich exterior of the hydrogel to increase the biodegradation of PPS and PLGA polymer to release TMZ. As expected, fast TMZ release from the T/PPS + TMZ hydrogel was observed when MMP2 + H_2_O_2_ (68.7%) and MMP9 + H_2_O_2_ (65.0%) were applied. Compared with the T/PPS + TMZ hydrogel, the T/PLGA + TMZ hydrogel without ROS responsiveness, which was immersed in MMP2 + H_2_O_2_ (47.3%) and MMP9 + H_2_O_2_ (46.7%), had almost the same release of TMZ in the presence of MMPs (Fig. 3B). Interestingly, neither the T/PPS + TMZ hydrogel nor the T/PLGA + TMZ hydrogel significantly improved the release of TMZ with H_2_O_2_ treatment alone. This may be due to the structure of hydrogel. The ROS-responsive PPS is located inside the hydrogel, and drug release was not significantly affected by PPS alone unless the T in the outer layer was degraded.

Next, MMP- and ROS-responsive T/PPS + TMZ hydrogel disassembly was assessed. Compared with the T/PLGA + TMZ hydrogel, the T/PPS + TMZ hydrogel soaked in PBS containing MMPs and H_2_O_2_ had little residual hydrogel after 5 days of immersion, indicating that more of the T/PPS + TMZ hydrogel was disassembled under the ROS and MMPs conditions (Fig. 3C). To further monitor hydrogel degradation, T/PPS and T/PLGA hydrogels were loaded with DiR to form T/PPS + DiR and T/PLGA + DiR hydrogels. Compared with the T/PLGA + DiR hydrogel in the presence of MMP2 + H_2_O_2_, the T/PPS + DiR hydrogel had a high dose-dependent loss of DiR fluorescence (Fig. 3D, E). The above results indicate that the T/PPS + TMZ hydrogel had an obvious ROS-responsive ability.

**Fig. 4:**
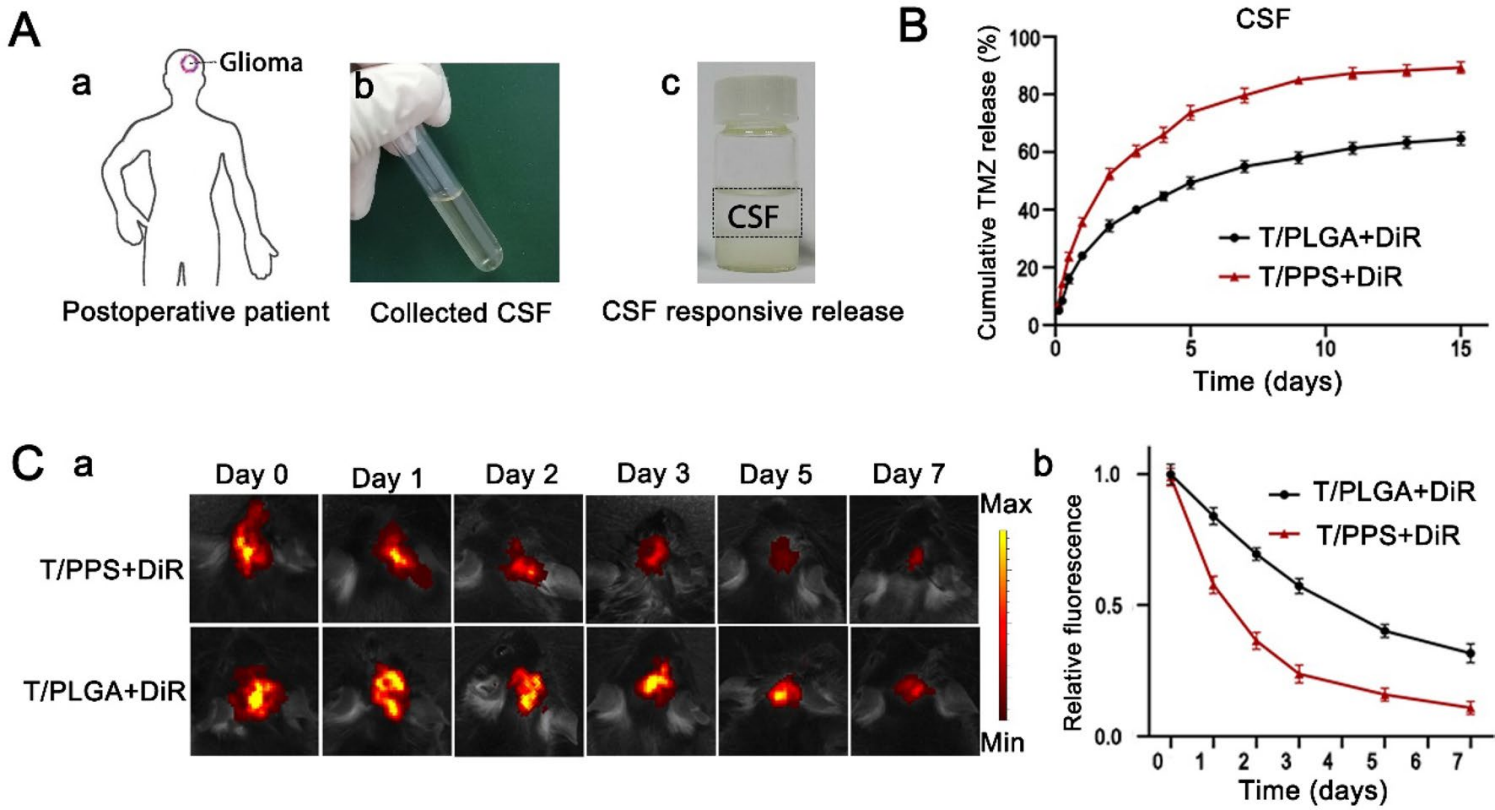
Responsive degradation of the T/PPS + TMZ hydrogel in a postoperative environment. **A** CSF was collected from glioma patients after the operation. **B** The release profile of the T/PPS + TMZ and T/PLGA + TMZ hydrogels. Data are presented as means ± SEM (n = 3). **C** Fluorescence intensity of the T/PPS + DiR hydrogel and T/PLGA + DiR hydrogel after injection into the surgical cavity of GL261-bearing mice at 0, 1, 2, 3, 5, and 7 days (**a**). Quantitative analysis of DiR fluorescence signals of mice at 0, 1, 2, 3, 5, and 7 days (**b**). Data are presented as the means ± SEM (n = 3)

To mimic the postsurgical microenvironment precisely, cerebrospinal fluid (CSF) from postsurgical glioma patients (Department of Neurosurgery, Affiliated Hospital of Xuzhou Medical University, Hospitalization Number: 1782969) was collected to test the release kinetics of TMZ (Fig. 4A). As shown in Fig. 4B, 89.3% TMZ was released from the T/PPS + TMZ hydrogel under CSF conditions, while only 62.3% TMZ was released from the T/PLGA + TMZ hydrogel under the same conditions. Furthermore, to monitor hydrogel disassembly in vivo, we investigated the ability of the T/PPS + DiR hydrogel to disassemble in vivo using a postsurgical glioma model. As shown in Fig. 4C, the animals were examined on days 1, 2, 3, 5, and 7 using the IVIS kinetic imaging system in vivo. As expected, the fluorescence signal of the T/PPS + DiR hydrogel decayed more rapidly than that of the T/PLGA + DiR hydrogel in the postsurgical environment. These results demonstrate that the T/PPS + DiR hydrogel could respond to the postsurgical environment and release the encapsulated drug.

### Cytotoxicity of T/PPS hydrogel

**Fig. 5:**
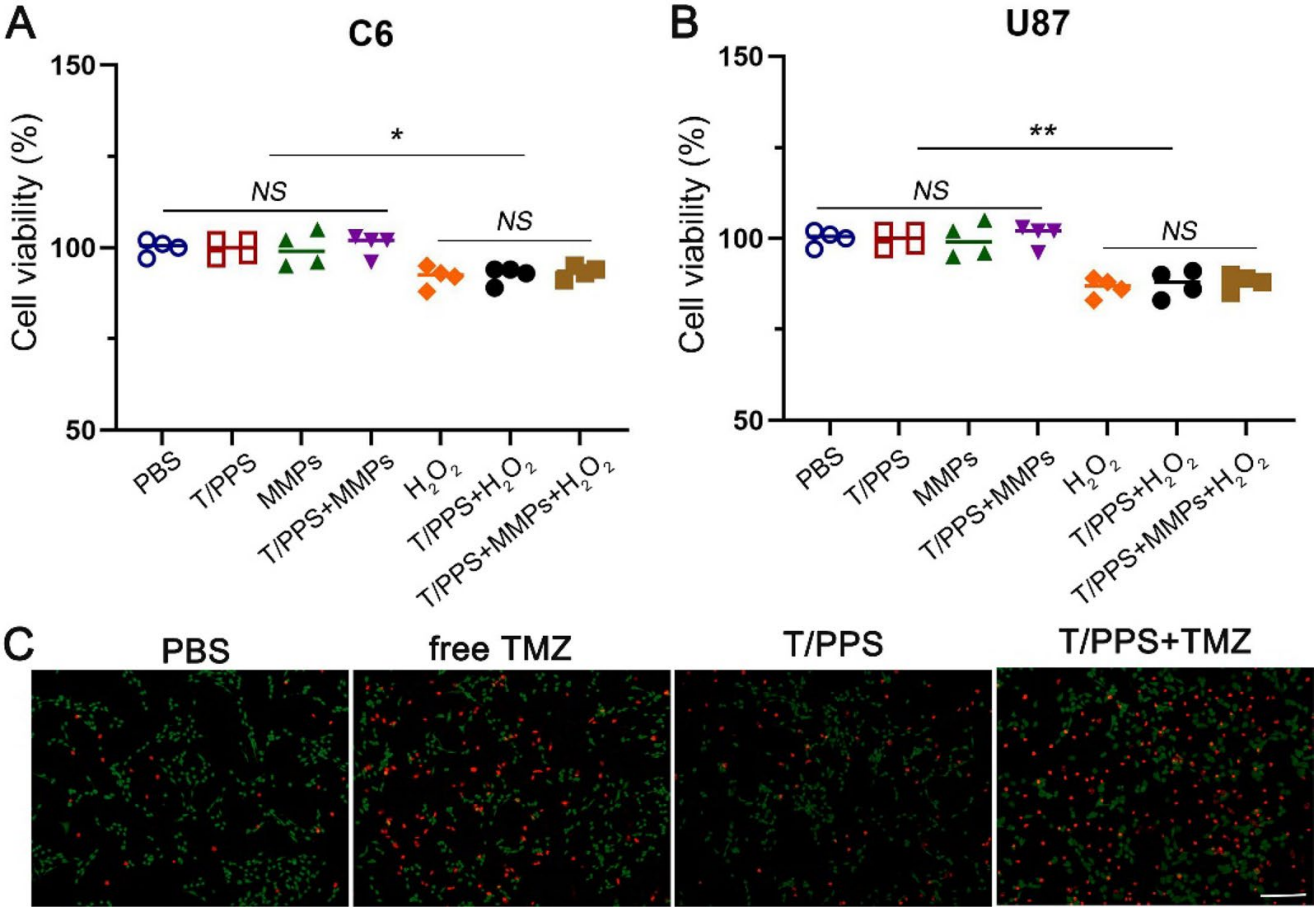
T/PPS hydrogels are biocompatible in vitro. **A**, **B** U87 and C6 glioma cells under different stimuli. Cell metabolic activity was determined by MTT assay after 24 h. Statistical values were calculated using one-way ANOVA. Data are presented as the means ± SEM. (n = 3). **C** Soaking solution of hydrogels was added to the medium of C6 glioma cells, and live/dead staining was performed after incubation for 72 h. Scale bar = 200 µm

Safety is paramount in the clinical application of biomaterials. Next, we investigated the cytotoxicity of different hydrogels to C6 and U87 glioma cells by MTT assay. As shown in Fig. 5A, the survival of C6 cells was hardly affected by the T/PPS hydrogel, suggesting that the T/PPS hydrogel had little cytotoxicity. Similar results were obtained when U87 cells were treated in the same way (Fig. 5B). Live/dead staining also did not show a significant loss of cell viability or changes in cell morphology in C6 glioma cells incubated with hydrogels (Fig. 5C). However, in the T/PLGA + TMZ and T/PPS + TMZ hydrogel groups, the intensity of red fluorescence increased. These results showed that the release of TMZ from the T/PLGA + TMZ and T/PPS + TMZ hydrogel groups led to the death of C6 glioma cells.

### Anti-glioma effect of T/PPS + TMZ hydrogel in vivo

**Fig. 6:**
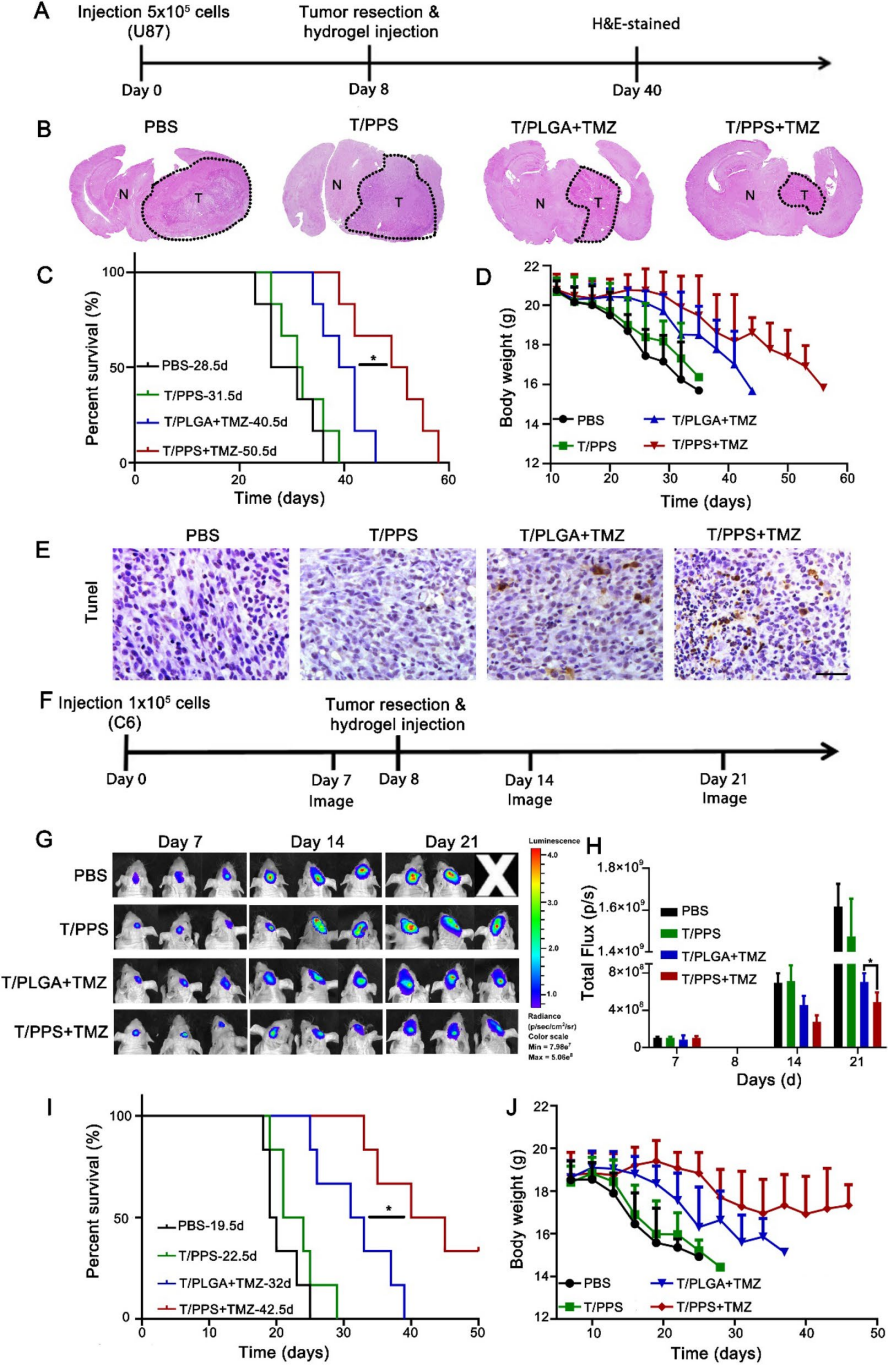
The T/PPS + TMZ hydrogel reduced recurrence in orthotopic U87 and C6-GFP-Luci tumour resection model mice. **A** Experimental design of the use of the T/PPS + TMZ hydrogel in orthotopic U87 tumour resection model mice. **B** Representative H&E-stained images of coronal brain sections of tumour-bearing mice. **C** Survival curves (n = 6) in different groups. Statistical values were calculated using one-way ANOVA. **D** Body weight change. Data are presented as the means ± SEM (n = 6). **E** TUNEL staining of coronal sections from mouse brains with orthotopic tumours. Scale bar = 50 µm. **F** Experimental design of the use of T/PPS + TMZ hydrogel in the orthotopic C6-Luci tumour resection model. **G**, **H** In vivo bioluminescence imaging (**G**) of C6-GFP-Luci tumours after resection and quantitative analysis (**H**) of the tumour-bearing mice at days 7, 14, and 21 after treatment. **I** Survival curves (n = 6) in different groups. Statistical values were calculated using one-way ANOVA. **J** Body weight change. Data are presented as the mean ± SEM (n = 6)

To further investigate whether the T/PPS + TMZ hydrogel can effectively inhibit recurrent glioma, an incomplete resection model of U87 glioma was established in BALB/c nude mice. On the 12th day after tumour implantation, the operation was performed, and the T/PPS + TMZ hydrogel was injected into the operation cavity. After treatment for 18 days, H&E staining was performed to confirm the therapeutic effects of the T/PPS + TMZ hydrogel (Fig. 6A). Compared with that in the PBS group, the size of the recurrent glioma T/PPS hydrogel group after surgery was almost the same volume because the T/PPS hydrogel had no side effects. Mice treated with T/PLGA + TMZ and the T/PPS + TMZ hydrogel delayed tumour recurrence, in which the T/PPS + TMZ hydrogel exhibited an anti-glioma efficacy superior to that of the T/PLGA + TMZ hydrogel, indicating that MMPs and ROS responsiveness could enhance the drug release of TMZ (Fig. 6B). As expected, the T/PPS + TMZ hydrogel performed best in extending the median survival (50.5 days), while the median survival of mice treated with PBS, T/PPS, and T/PLGA + TMZ was 28.5, 31.5, and 40.5 days, respectively (Fig. 6C). This variation was also reflected in the body weight changes of tumour-bearing mice. The body weights of mice in the T/PPS + TMZ group decreased slowly, while the other groups lost weight rapidly (Fig. 6D). To further evaluate the anti-glioma effect of the T/PPS + TMZ hydrogel, apoptosis detection (TUNEL) was used to detect the apoptosis of glioma cells. As shown in Fig. 6E, the highest proportion of apoptotic cells was observed in the T/PPS + TMZ hydrogel group among the other treatment groups. These results suggest that MMPs- and ROS-responsive T/PPS + TMZ hydrogels could enhance TMZ therapy to induce apoptotic cell death in glioma.

Furthermore, a TMZ-resistant C6-Luci glioma orthotopic model was developed to further evaluate the inhibition glioma recurrence efficiency of the T/PPS + TMZ hydrogel. An incomplete tumour operation was performed 8 days after implantation, and the T/PPS + TMZ hydrogels were injected into the surgical cavity (Fig. 6F). The fluorescence intensity of C6-Luci gliomas was measured to evaluate recurrent glioma (Fig. 6G, H). Glioma recurred quickly after the operation, and glioma growth was faster in the PBS and T/PPS hydrogel groups. The T/PPS + TMZ and T/PLGA + TMZ hydrogel groups showed better anti-glioma efficacy with a lower intensity of bioluminescence fluorescence than the PBS and T/PPS groups, demonstrating that in situ injection of TMZ-embedded hydrogel could inhibit residual glioma cell growth. Moreover, compared with the T/PLGA + TMZ hydrogel group, the T/PPS + TMZ hydrogel group had lower bioluminescence fluorescence intensity, suggesting that the T/PPS + TMZ hydrogel group effectively reduced tumour regrowth. In addition, we monitored weight changes and median survival time in mice. As shown in Fig. 6I, the median survival of mice treated with PBS, T/PPS hydrogel, T/PLGA + TMZ hydrogel and T/PPS + TMZ hydrogel was 19.5, 22.5, 32 and 50.5 days, respectively. The median survival of the mice treated with the T/PPS + TMZ hydrogel was the longest, and the mouse body weight decreased the most slowly among all groups (Fig. 6J). Altogether, these results further demonstrate that the T/PPS + TMZ hydrogel with MMPs and ROS-responsiveness ability improved the inhibition efficacy of postoperative glioma recurrence.

### Biocompatibility in vivo

**Fig. 7:**
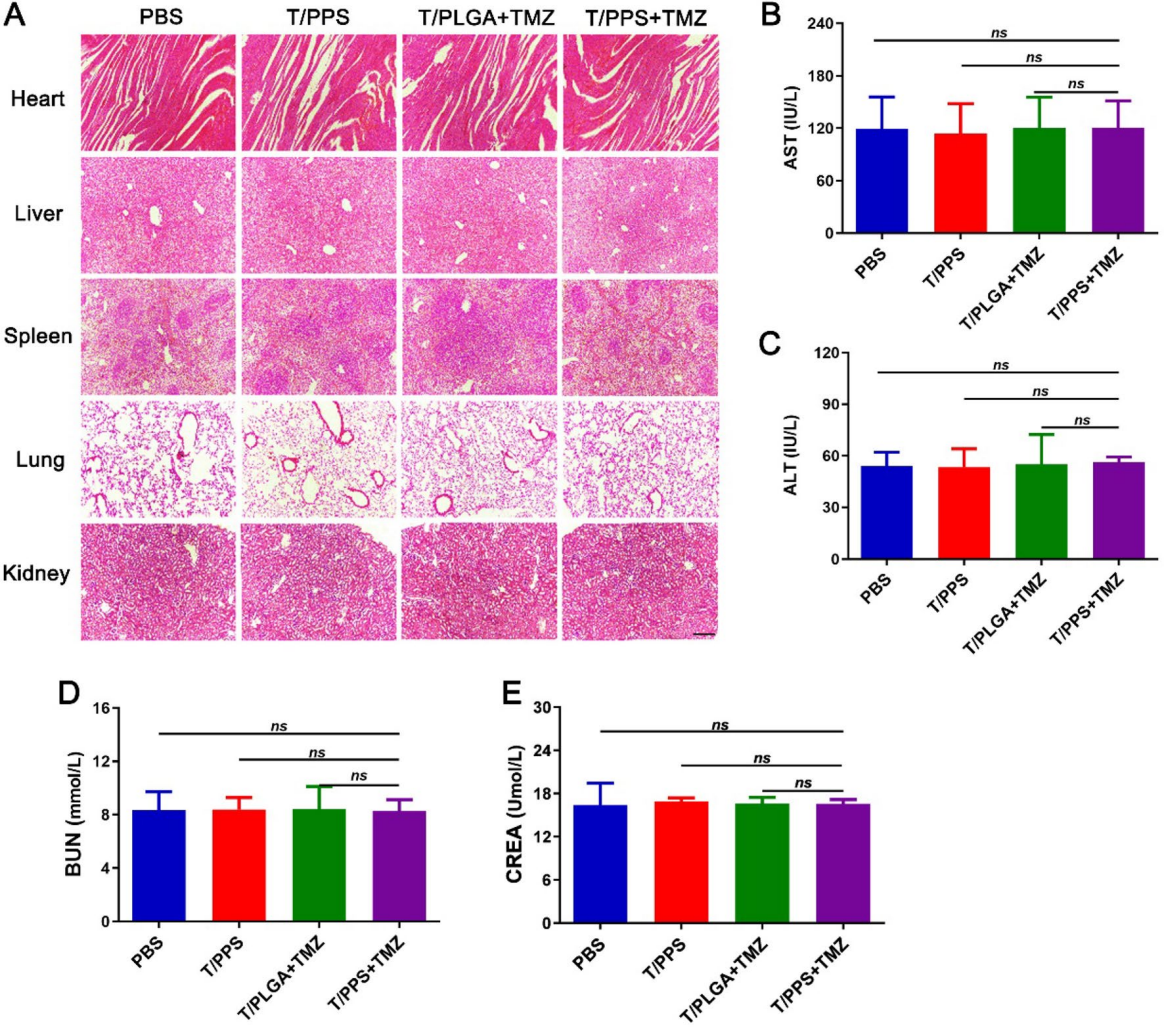
Systemic toxicity analysis. **A** H&E staining of heart, liver, spleen, lung and kidney from mice in different groups. The scale bar = 200 µm. **B**–**E** Detection of liver and kidney biochemical indices, including aspartate aminotransferase (AST), alanine aminotransferase (ALT), blood urea nitrogen (BUN), and creatinine (CREA). Statistical values were calculated using one-way ANOVA. Data are presented as the mean ± SEM (n = 3)

For safety evaluation, the potential toxicity of the hydrogels was systematically investigated in vivo. After 10 days of treatment, the main organs, including the liver, spleen, kidney, heart, and lung, were collected and sliced into sections for H&E staining. As shown in Fig. 7A, no apparent histological abnormality or lesion was observed among the groups, indicating that the T/PPS hydrogel had no apparent toxicity in vivo. The results of the blood biochemical analysis were likewise similar, demonstrating that the T/PPS hydrogel did not induce significant damage to the liver (Fig. 7B, C) or kidneys (Fig. 7D, E). Taken together, these results demonstrate that the T/PPS + TMZ hydrogel showed excellent biocompatibility in vivo*.*

## Discussion

Glioma is associated with the deterioration and evolution of neuroepithelial cells and accounts for 45% of primary brain tumours [10, 45]. Due to the aggressive growth of glioma and peripheral brain tissue and because many tumours are located in important functional areas, such as the brainstem, thalamus and central sulcus, complete surgical removal of malignant glioma is impossible, and tumour recurrence frequently occurs [46, 47]. In fact, due to the existence of the BBB, limiting systemic toxicity and chemotherapy resistance, the efficacy of traditional cytotoxic drugs was severely blunted. In this case, local drug delivery offers a desirable alternative. However, no other topical treatments based on hydrogels have been approved by the FDA since Gliadel® entered the market in 1997. Considering the potential of hydrogels, this phenomenon was perplexing.

To obtain ideal drug-loaded hydrogels for treating recurrent glioma, many factors must be taken into account: (1) anticancer drugs with good performance. Many drugs have received attention in preclinical research, such as gemcitabine, trimethoprim, and 5-fluorouracil. However, TMZ is the treatment of choice for patients with glioma, so the local delivery of TMZ and prevention of chemoresistance should be the focus. (2) Excellent injectability and adhesiveness should be considered. Due to the anatomy and structure of the resection cavity, semisolid hydrogels with a certain degree of fluidity would be preferable. (3) Degradability and biocompatibility. The hydrogel material should have good safety and be biodegradable to avoid repeated brain operations and use as a scaffold for tumour growth. Thus, natural or artificial materials with nontoxicity and good solubility were the primary targets.

For the reasons stated above, our study selected a Tm hydrogel to prepare a biocompatible, glioma microenvironment-responsive hydrogel. To explore the characteristics of the drug-loaded hydrogel, we analysed the morphological features, rheological behaviours and drug release. The results showed that the T/PPS + TMZ hydrogel had good biocompatibility and improved the release of TMZ in the glioma microenvironment.

Simultaneously, we explored the effect of inhibiting glioma cells in vitro. The MTT assay verified that the novel hydrogel T had no obvious cytotoxicity, indicating that the T/PPS + TMZ hydrogel had highly favourable safety. In addition, the T/PPS + TMZ hydrogel demonstrated excellent performance in the live/dead assay, which indicated that the T/PPS + TMZ hydrogel has an excellent cell-killing effect against tumour cells.

Furthermore, we constructed in situ glioma models to investigate the antitumour effect of the T/PPS + TMZ hydrogel in vivo. For the subcutaneous U87 in situ glioma model, H&E sections of U87 cell glioma in situ were prepared to detect the antitumour effect, and survival and weight curves were drawn. The results showed that the T/PPS + TMZ group performed best among all groups, the median survival period of nude mice in the T/PPS + TMZ group was prolonged significantly, and their body weight decreased slowly. For the C6-GFP in situ glioma model, after the tumour was confirmed by the IVIS Spectrum In Vivo Imaging System on the 7th day after tumour implantation, the glioma was resected under the microscope, and different groups of hydrogels were injected into the cavity. The tumours were also assessed on days 14 and 21. The results showed that the fluorescence intensity of the T/PPS + TMZ group was significantly lower than that of the other groups, and the experimental group had a longer median survival. Thus, we verified that the T/PPS + TMZ hydrogel more effectively inhibits tumour proliferation and increases tumour apoptosis.

In summary, we successfully constructed a highly biocompatible, glioma microenvironment-responsive T/PPS + TMZ hydrogel. The T/PPS + TMZ hydrogel enhanced the potential efficacy of TMZ in vitro and in vivo and exhibited a robust effect against postoperative glioma recurrence. Even so, further clinical trials are warranted to support this method.

## Conclusion

In summary, we successfully developed an injectable MMPs- and ROS-responsive hydrogel. These hydrogels exhibited postoperative environmental responsiveness and achieved sustained TMZ release in the surgical cavity. The in vitro drug release study confirmed this property of the T/PPS + TMZ hydrogel. Using CSF from postsurgical glioma patients and a postsurgical glioma model further demonstrated that the T/PPS hydrogel had MMPs-/ROS-responsive ability in a postoperative environment. The antiglioma effects in the incomplete operation models of C6 and U87 glioma indicated that the T/PPS + TMZ hydrogel effectively inhibited postsurgical glioma recurrence while minimizing systemic toxicity. Overall, the T/PPS + TMZ hydrogel significantly improved the effects of TMZ and may be utilized as a potential strategy to decrease the recurrence rate of glioma after surgery.

## Supplementary Information

**Additional file 1: Figure S1.** (A) Specification of the biopsy punch. (B) Expanding previous burr hole to expose brain and tumor tissue (a). Resection the tumor by a 2 mm biopsy punch (b). Resection cavity (c). Hydrogels were injected into the resection cavity (d). **Figure S2.** Expression of MMP2 and MMP9 in Sham/Resection group in vivo*.* **Figure S3.** (A) Representative images of ROS staining in Sham and Resection group in vivo using green fluorescent probe DCFH-DA. Scale bar = 300 µm. (B) The expressions of ROS were analyzed quantitatively using integral optical density (IOD) analysis.

## Data Availability

All data generated or analysed during this study are included in this manuscript and its Additional file.

## Abbreviations

TMZ: Temozolomide
PPS: Poly (propylene sulfide)
MMPs: Matrix metalloproteinases
T: Triglycerol monostearate
ROS: Reactive oxygen species
BBB: Blood–brain barrier
MGMT: *O*6-Mehtylguanine-DNA methyl transferase
PLGA: Poly (lactic-co-glycolic acid)
PDI: Polydispersity
CSF: Cerebrospinal fluid

## References

[1] Ito H, Nakashima H, Chiocca EA (2019). Molecular responses to immune checkpoint blockade in glioblastoma. Nat Med.

[2] Gao H, Jiang X (2013). Progress on the diagnosis and evaluation of brain tumors. Cancer Imaging.

[3] Castaneda CA, Casavilca S, Orrego E, Garcia-Corrochano P, Deza P, Heinike H, Castillo M, Belmar-Lopez C, Ojeda L (2015). Glioblastoma: molecular analysis and its clinical implications. Rev Peru Med Exp Salud Publica.

[4] Rich JN, Bigner DD (2004). Development of novel targeted therapies in the treatment of malignant glioma. Nat Rev Drug Discov.

[5] Liu EK, Sulman EP, Wen PY, Kurz SC (2020). Novel therapies for glioblastoma. Curr Neurol Neurosci Rep.

[6] Lin L, Cai J, Jiang C (2017). Recent advances in targeted therapy for glioma. Curr Med Chem.

[7] Templeton A, Hofer S, Töpfer M, Sommacal A, Fretz C, Cerny T, Gillessen S (2008). Extraneural spread of glioblastoma—report of two cases. Onkologie.

[8] Ahmed R, Oborski MJ, Hwang M, Lieberman FS, Mountz JM (2014). Malignant gliomas: current perspectives in diagnosis, treatment, and early response assessment using advanced quantitative imaging methods. Cancer Manag Res.

[9] Nitta M, Muragaki Y, Maruyama T, Ikuta S, Komori T, Maebayashi K, Iseki H, Tamura M, Saito T, Okamoto S, Chernov M, Hayashi M, Okada Y (2015). Proposed therapeutic strategy for adult low-grade glioma based on aggressive tumor resection. Neurosurg Focus.

[10] Sharma RR, Singh DP, Pathak A, Khandelwal N, Sehgal CM, Kapoor R, Ghoshal S, Patel FD, Sharma SC (2003). Local control of high-grade gliomas with limited volume irradiation versus whole brain irradiation. Neurol India.

[11] Kadiyala P, Li D, Nuñez FM, Altshuler D, Doherty R, Kuai R, Yu M, Kamran N, Edwards M, Moon JJ, Lowenstein PR, Castro MG, Schwendeman A (2019). High-density lipoprotein-mimicking nanodiscs for chemo-immunotherapy against glioblastoma multiforme. ACS Nano.

[12] Zhang J, Stevens MF, Bradshaw TD (2012). Temozolomide: mechanisms of action, repair and resistance. Curr Mol Pharmacol.

[13] Ge X, Pan MH, Wang L, Li W, Jiang C, He J, Abouzid K, Liu LZ, Shi Z, Jiang BH (2018). Hypoxia-mediated mitochondria apoptosis inhibition induces temozolomide treatment resistance through miR-26a/Bad/Bax axis. Cell Death Dis.

[14] Yan Y, Xu Z, Dai S, Qian L, Sun L, Gong Z (2016). Targeting autophagy to sensitive glioma to temozolomide treatment. J Exp Clin Cancer Res.

[15] Ward SM, Skinner M, Saha B, Emrick T (2018). Polymer-temozolomide conjugates as therapeutics for treating glioblastoma. Mol Pharm.

[16] Yang B, Ma YB, Chu SH (2018). Silencing SATB1 overcomes temozolomide resistance by downregulating MGMT expression and upregulating SLC22A18 expression in human glioblastoma cells. Cancer Gene Ther.

[17] Jiang G, Li LT, Xin Y, Zhang L, Liu YQ, Zheng JN (2012). Strategies to improve the killing of tumors using temozolomide: targeting the DNA repair protein MGMT. Curr Med Chem.

[18] Hong LTA, Kim YM, Park HH, Hwang DH, Cui Y, Lee EM, Yahn S, Lee JK, Song SC, Kim BG (2017). An injectable hydrogel enhances tissue repair after spinal cord injury by promoting extracellular matrix remodeling. Nat Commun.

[19] Norouzi M, Nazari B, Miller DW (2016). Injectable hydrogel-based drug delivery systems for local cancer therapy. Drug Discov Today.

[20] Bastiancich C, Vanvarenberg K, Ucakar B, Pitorre M, Bastiat G, Lagarce F, Préat V, Danhier F (2016). Lauroyl-gemcitabine-loaded lipid nanocapsule hydrogel for the treatment of glioblastoma. J Control Release.

[21] Liu H, Shi X, Wu D, Kahsay Khshen F, Deng L, Dong A, Wang W, Zhang J (2019). Injectable, biodegradable, thermosensitive nanoparticles-aggregated hydrogel with tumor-specific targeting, penetration, and release for efficient postsurgical prevention of tumor recurrence. ACS Appl Mater Interfaces.

[22] Wu H, Song L, Chen L, Zhang W, Chen Y, Zang F, Chen H, Ma M, Gu N, Zhang Y (2018). Injectable magnetic supramolecular hydrogel with magnetocaloric liquid-conformal property prevents post-operative recurrence in a breast cancer model. Acta Biomater.

[23] Yang WJ, Zhou P, Liang L, Cao Y, Qiao J, Li X, Teng Z, Wang L (2018). Nanogel-incorporated injectable hydrogel for synergistic therapy based on sequential local delivery of combretastatin-A4 phosphate (CA4P) and doxorubicin (DOX). ACS Appl Mater Interfaces.

[24] Bastiancich C, Bozzato E, Luyten U, Danhier F, Bastiat G, Préat V (2019). Drug combination using an injectable nanomedicine hydrogel for glioblastoma treatment. Int J Pharm.

[25] Deng H, Dong A, Song J, Chen X (2019). Injectable thermosensitive hydrogel systems based on functional PEG/PCL block polymer for local drug delivery. J Control Release.

[26] Townsend JM, Beck EC, Gehrke SH, Berkland CJ, Detamore MS (2019). Flow behavior prior to crosslinking: the need for precursor rheology for placement of hydrogels in medical applications and for 3D bioprinting. Prog Polym Sci.

[27] Talebian S, Mehrali M, Taebnia N, Pennisi CP, Kadumudi FB, Foroughi J, Hasany M, Nikkhah M, Akbari M, Orive G, Dolatshahi-Pirouz A (2019). Self-healing hydrogels: the next paradigm shift in tissue engineering?. Adv Sci.

[28] Loebel C, Rodell CB, Chen MH, Burdick JA (2017). Shear-thinning and self-healing hydrogels as injectable therapeutics and for 3D-printing. Nat Protoc.

[29] Han J, Cui Y, Han X, Liang C, Liu W, Luo D, Yang D (2020). Super-soft DNA/dopamine-grafted-dextran hydrogel as dynamic wire for electric circuits switched by a microbial metabolism process. Adv Sci.

[30] Grim JC, Brown TE, Aguado BA, Chapnick DA, Viert AL, Liu X, Anseth KS (2018). A reversible and repeatable thiol-ene bioconjugation for dynamic patterning of signaling proteins in hydrogels. ACS Cent Sci.

[31] Fourniols T, Randolph LD, Staub A, Vanvarenberg K, Leprince JG, Préat V, des Rieux A, Danhier F (2015). Temozolomide-loaded photopolymerizable PEG-DMA-based hydrogel for the treatment of glioblastoma. J Control Release.

[32] Zhao G, Jia J, Wang L, Zhang Y, Yang H, Lu Y, Yu R, Liu H, Zhu Y (2020). Local delivery of minocycline and vorinostat targets the tumor microenvironment to inhibit the recurrence of glioma. Onco Targets Ther.

[33] Chao Y, Xu L, Liang C, Feng L, Xu J, Dong Z, Tian L, Yi X, Yang K, Liu Z (2018). Combined local immunostimulatory radioisotope therapy and systemic immune checkpoint blockade imparts potent antitumour responses. Nat Biomed Eng.

[34] Bastiancich C, Danhier P, Préat V, Danhier F (2016). Anticancer drug-loaded hydrogels as drug delivery systems for the local treatment of glioblastoma. J Control Release.

[35] Gupta MK, Martin JR, Werfel TA, Shen T, Page JM, Duvall CL (2014). Cell protective, ABC triblock polymer-based thermoresponsive hydrogels with ROS-triggered degradation and drug release. J Am Chem Soc.

[36] Zhao Z, Shen J, Zhang L, Wang L, Xu H, Han Y, Jia J, Lu Y, Yu R, Liu H (2020). Injectable postoperative enzyme-responsive hydrogels for reversing temozolomide resistance and reducing local recurrence after glioma operation. Biomater Sci.

[37] Liu H, Xie Y, Zhang Y, Cai Y, Li B, Mao H, Liu Y, Lu J, Zhang L, Yu R (2017). Development of a hypoxia-triggered and hypoxic radiosensitized liposome as a doxorubicin carrier to promote synergetic chemo-/radio-therapy for glioma. Biomaterials.

[38] Hua L, Wang Z, Zhao L, Mao H, Wang G, Zhang K, Liu X, Wu D, Zheng Y, Lu J, Yu R, Liu H (2018). Hypoxia-responsive lipid-poly-(hypoxic radiosensitized polyprodrug) nanoparticles for glioma chemo- and radiotherapy. Theranostics.

[39] Bianco J, Bastiancich C, Joudiou N, Gallez B, Des Rieux A, Danhier F (2017). Novel model of orthotopic U-87 MG glioblastoma resection in athymic nude mice. J Neurosci Methods.

[40] Müller-Quernheim J (2011). MMPs are regulatory enzymes in pathways of inflammatory disorders, tissue injury, malignancies and remodelling of the lung. Eur Respir J.

[41] Niethammer P, Grabher C, Look AT, Mitchison TJ (2009). A tissue-scale gradient of hydrogen peroxide mediates rapid wound detection in zebrafish. Nature.

[42] Hu YP, Peng YB, Zhang YF, Wang Y, Yu WR, Yao M, Fu XJ (2017). Reactive oxygen species mediated prostaglandin E(2) contributes to acute response of epithelial injury. Oxid Med Cell Longev.

[43] Joshi N, Yan J, Levy S, Bhagchandani S, Slaughter KV, Sherman NE, Amirault J, Wang Y, Riegel L, He X, Rui TS, Valic M, Vemula PK, Miranda OR, Levy O, Gravallese EM, Aliprantis AO, Ermann J, Karp JM (2018). Towards an arthritis flare-responsive drug delivery system. Nat Commun.

[44] Gajanayake T, Olariu R, Leclère FM, Dhayani A, Yang Z, Bongoni AK, Banz Y, Constantinescu MA, Karp JM, Vemula PK, Rieben R, Vögelin E (2014). A single localized dose of enzyme-responsive hydrogel improves long-term survival of a vascularized composite allograft. Sci Transl Med.

[45] Ajaz M, Jefferies S, Brazil L, Watts C, Chalmers A (2014). Current and investigational drug strategies for glioblastoma. Clin Oncol (R Coll Radiol).

[46] Mrugala MM, Chamberlain MC (2008). Mechanisms of disease: temozolomide and glioblastoma—look to the future. Nat Clin Pract Oncol.

[47] Quinn JA, Desjardins A, Weingart J, Brem H, Dolan ME, Delaney SM, Vredenburgh J, Rich J, Friedman AH, Reardon DA, Sampson JH, Pegg AE, Moschel RC, Birch R, McLendon RE, Provenzale JM, Gururangan S, Dancey JE, Maxwell J, Tourt-Uhlig S, Herndon JE, Bigner DD, Friedman HS (2005). Phase I trial of temozolomide plus O6-benzylguanine for patients with recurrent or progressive malignant glioma. J Clin Oncol.

