# Supplementary material for: Multi-responsive nanofibers composite gel for local drug delivery to inhibit recurrence of glioma after operation
Source: J Nanobiotechnology. 2021 Jul 3;19:198. doi: 10.1186/s12951-021-00943-z (PMC8255008; doi:10.1186/s12951-021-00943-z)
Supplement: Supplementary file 1 — Additional file 1: Figure S1. (A) Specification of the biopsy punch. (B) Expanding previous burr hole to expose brain and tumor tissue (a). Resection the tumor by a 2 mm biopsy punch (b). Resection cavity (c). Hydrogels were injected into the resection cavity (d). Figure S2. Expression of MMP2 and MMP9 in Sham/Resection group in vivo. Figure S3. (A) Representative images of ROS staining in Sham and Resection group in vivo using green fluorescent probe DCFH-DA. Scale bar = 300 µm. (B) The expressions of ROS were analyzed quantitatively using integral optical density (IOD) analysis. [file 12951_2021_943_MOESM1_ESM.docx]

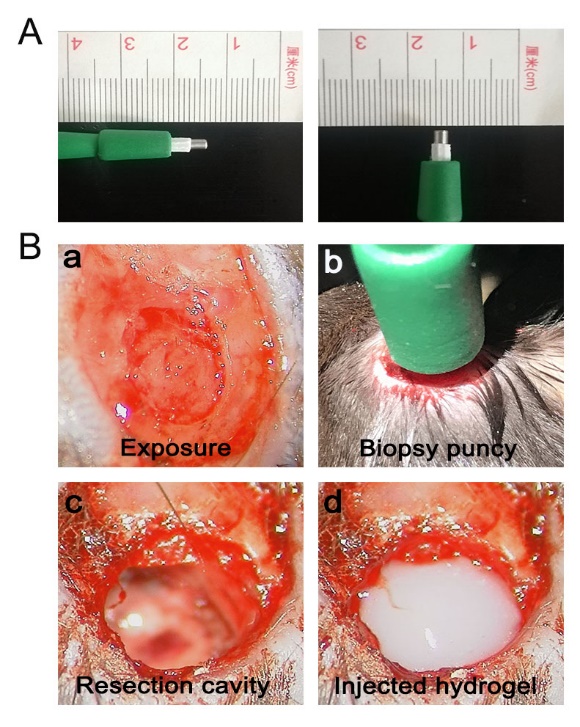
Figure S1. (A) Specification of the biopsy punch. (B) Expanding previous burr hole to expose brain and tumor tissue (a). Resection the tumor by a 2 mm biopsy punch (b). Resection cavity (c). Hydrogels were injected into the resection cavity (d).


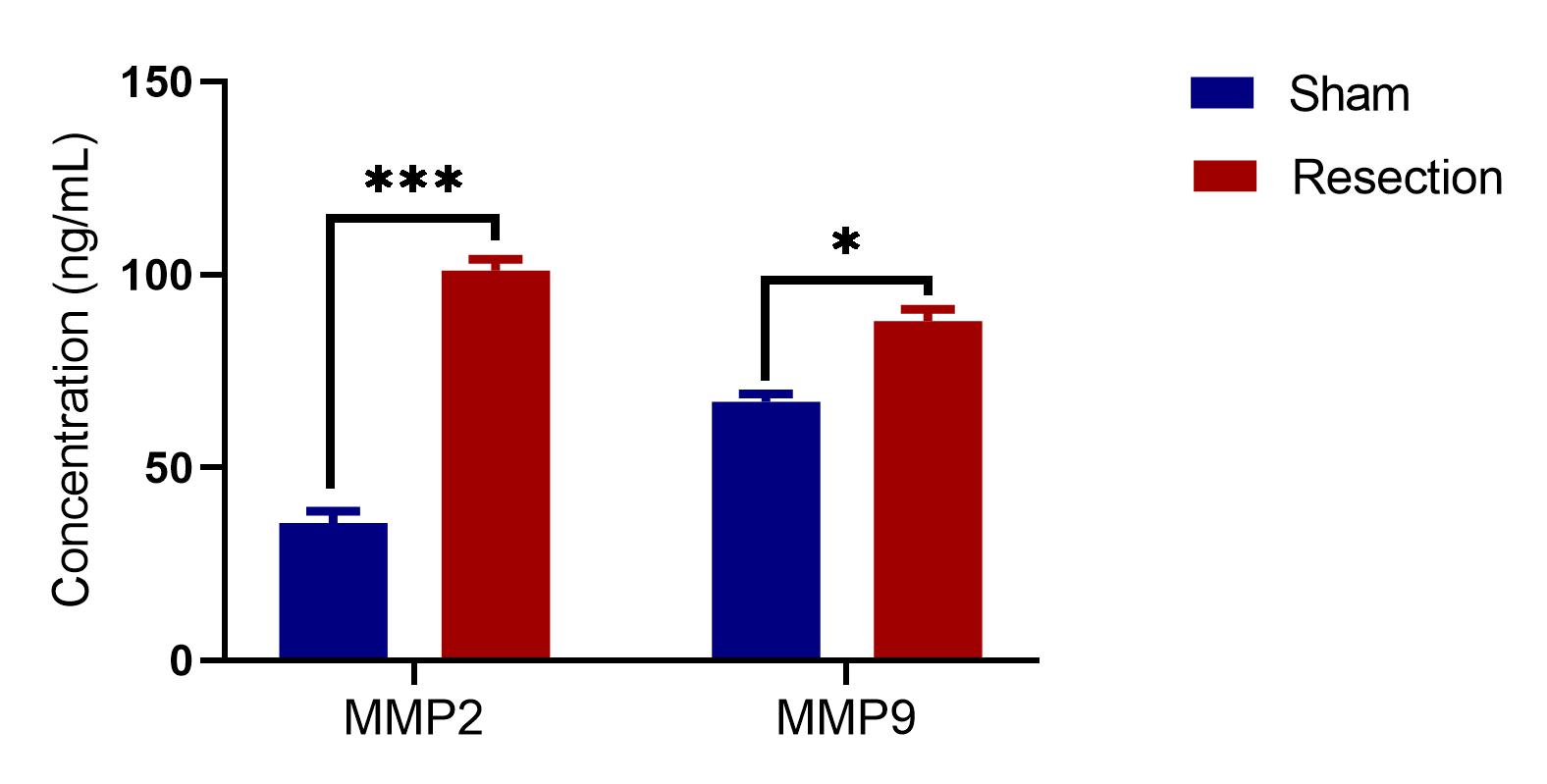


Figure S2. Expression of MMP2 and MMP9 in Sham/Resection group *in vivo.*

Figure
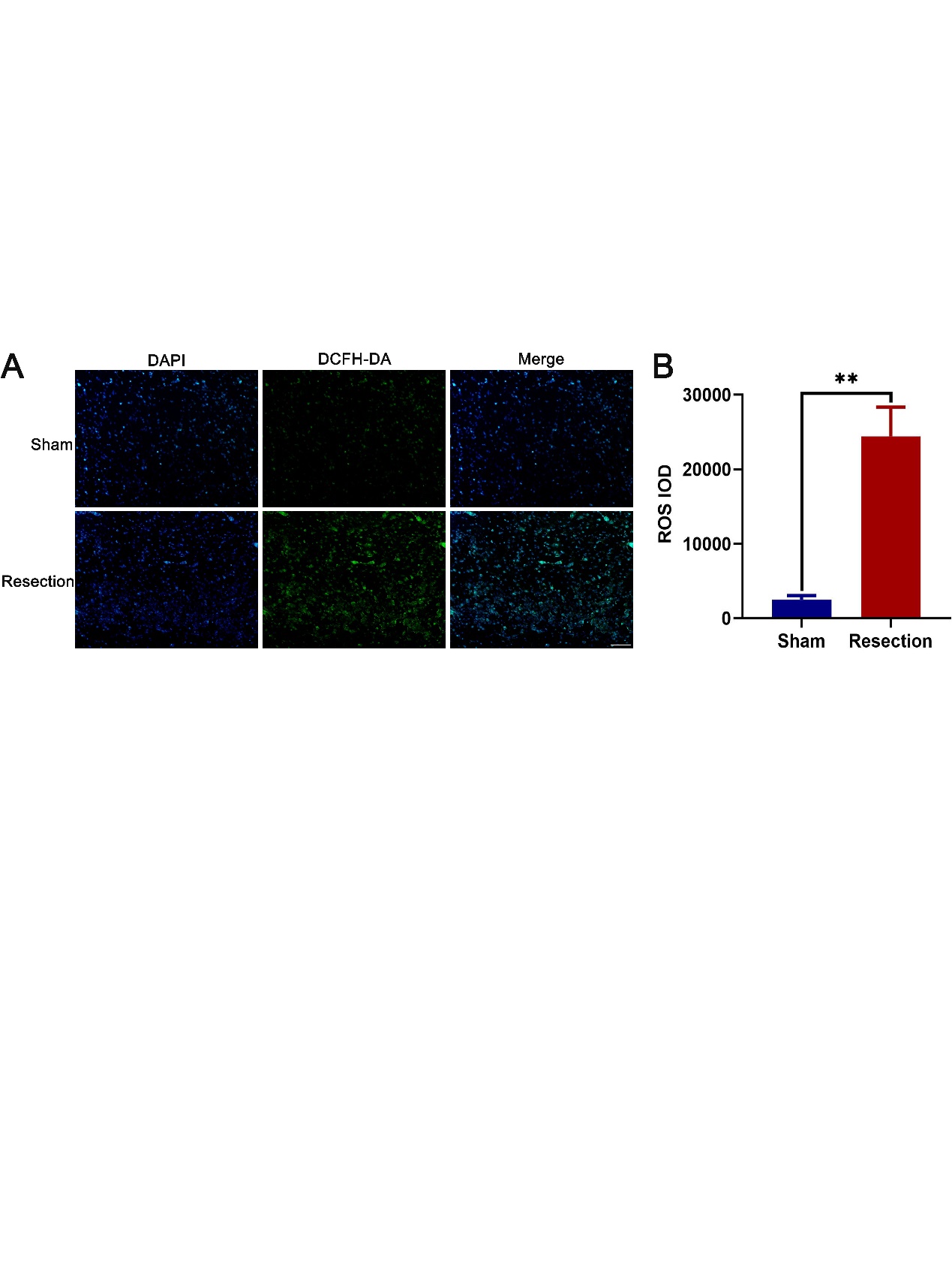
 S3. (A) Representative images of ROS staining in Sham and Resection group *in vivo* using green fluorescent probe DCFH-DA. Scale bar = 300 µm. (B) The expressions of ROS were analyzed quantitatively using integral optical density (IOD) analysis.
